# 

**Published:** 1883-03

**Authors:** 


MARCH, 1883.

THIRTIETH YEAR.

### HALL'S JOURNAL OF HEALTH

Guaranteed Circulation 200,000 Copies in 12 Months.

*E. H. GIBBS, A.M., M.D., Editor,*

No. 135 EIGHTH STREET (Near Broadway), NEW YORK.

TERMS: $1 a Year. Single number, 10 cts.

